# Sentiment Analysis of Children and Youth Literature: Is There a Pollyanna Effect?

**DOI:** 10.3389/fpsyg.2020.574746

**Published:** 2020-09-24

**Authors:** Arthur M. Jacobs, Berenike Herrmann, Gerhard Lauer, Jana Lüdtke, Sascha Schroeder

**Affiliations:** ^1^Department of Experimental and Neurocognitive Psychology, Freie Universität Berlin, Berlin, Germany; ^2^Center for Cognitive Neuroscience Berlin (CCNB), Freie Universität Berlin, Berlin, Germany; ^3^Digital Humanities Lab, Universität Basel, Basel, Switzerland; ^4^Educational Psychology, University of Göttingen, Göttingen, Germany

**Keywords:** Pollyanna effect, positivity superiority effect, sentiment analysis, *SentiArt*, neurocognitive poetics, affective-aesthetic potential, digital humanities, children and youth literature

## Abstract

If the words of natural human language possess a universal positivity bias, as assumed by Boucher and Osgood’s (1969) famous Pollyanna hypothesis and computationally confirmed for large text corpora in several languages (Dodds et al., 2015), then children and youth literature (CYL) should also show a Pollyanna effect. Here we tested this prediction applying an unsupervised vector space model-based sentiment analysis tool called *SentiArt* (Jacobs, 2019) to two CYL corpora, one in English (372 books) and one in German (500 books). Pitching our analysis at the sentence level, and assessing semantic as well as lexico-grammatical information, both corpora show the Pollyanna effect and thus add further evidence to the universality hypothesis. The results of our multivariate sentiment analyses provide interesting testable predictions for future scientific studies of literature.

*“The 7-year-old child is perfectly familiar with both PRETTY and UGLY but he uses the former much more frequently than the latter.”* Boucher and Osgood(1969, p. 7).

## Introduction

In 1969 Boucher and Osgood presented influential evidence for the idea that “humans tend to look on (and talk about) the bright side of life” and coined this phenomenon the “Pollyanna hypothesis,” i.e., a universal human tendency to use evaluatively positive words more frequently, diversely and facilely than evaluatively negative words^1^. About 50 years and many technological advances later – especially in natural language processing (NLP), computational linguistics and machine learning methods – Dodds et al.(2015, p. 6) presented extensive cross-cultural data based on large-scale macroanalytic, univariate sentiment analyses of multi-lingual text corpora that support the hypothesis. They concluded their study with: “Overall, our major scientific finding is that when experienced in isolation and weighted properly according to use, words, which are the atoms of human language, present an emotional spectrum with a universal, self-similar positive bias. We emphasize that this apparent linguistic encoding of our social nature is a system-level property, and in no way asserts all natural texts will skew positive… or diminishes the salience of negative states (Forgas, 2013). Going forward, our word happiness assessments should be periodically repeated and carried out for new languages, tested on different demographics, and expanded to phrases both for the improvement of hedonometric instruments and to chart the dynamics of our collective social self.” In a similar vein, Greene(2017, p.12) found hints to a positivity bias in his summary of text analyses of the “corpus of the canon of western literature” concluding “that even though canonical literature from Homer to Hemmingway addresses death, war, heartache and tragedy, the overall cultural preoccupations of the western canon over history have been largely positive.”

Such a textual positivity bias has measurable consequences for text processing and reading behavior known as the *positivity superiority effect*, i.e., the observation that in many word recognition tasks positive words yield faster response times than neutral or negative ones (Lüdtke and Jacobs, 2015; for review, see Jacobs et al., 2015). This effect, which has also been observed in 6–12 year old children (Sylvester et al., 2016), is usually explained with the informational density hypothesis (Ashby and Isen, 1999; Kuchinke et al., 2005; Unkelbach et al., 2010). It claims that positive information is generally processed faster on grounds of subjective exposure frequency, i.e., the experienced frequency with which positive information is internally activated in memory. Taking subjective exposure frequency as a proxy for higher informational density of lexical representations of positive words thus would cause them to be processed faster because they are better elaborated and interconnected in memory. Indeed, there is neurocomputational evidence for the idea that positive words provide more and denser semantic long-term associations than neutral or negative ones (Hofmann and Jacobs, 2014) which has been related to the hippocampus being more generally involved in the processing of positive affect (Hofmann and Kuchinke, 2015).

## The Present Study

Both Dodds et al.’s (2015) and Greene’s (2017) studies used texts for adults. Inspired by the above citation from Boucher and Osgood (1969) and previous behavioral studies from our group also supporting the Pollyanna hypothesis (Sylvester et al., 2016), here we were interested in finding out to what extent international texts of children and youth literature (CYL) also show the Pollyanna effect, including the book who’s protagonist coined the effect (Porter, 1915). For this purpose, we submitted Porter’s book, as well as 372 English and 500 German books representing CYL, to a computational sentiment analysis using the empirically well validated *SentiArt* tool which is based on (semantic) vector space models/VSM (Jacobs, 2019; Jacobs and Kinder, 2019).

Given that so far the *SentiArt* tool was only used for the analysis of rating and reading data of adult persons, we first cross-validated it with valence rating data from the *kidBawl* study (Sylvester et al., 2016). We then report the results of the computational sentiment for Porter’s (1915) book “Pollyanna Grows Up” before applying *SentiArt* to two large CYL corpora in both English and German.

## Corpora

The textual data used in this study come from two published corpora, the Gutenberg Literary English Corpus (GLEC; Jacobs, 2018b) and the (German) childLex corpus (Schroeder et al., 2015). Since both have been extensively described in the aforementioned papers, here we just give a brief summary of GLEC-CYL and childLex. The GLEC-CYL corpus is a subset of GLEC, containing 372 books from 25 different authors such as Beatrix Potter, Lyman Frank Baum or RM Ballantyne. For copyright reasons this corpus contains only books published before 1952. In contrast, the 500 books in the German childLex corpus mainly contain post-war and contemporary exemplars such as the seven books from the Harry Potter series (e.g., Rowling, 1997) and include a nice mix of texts by a large variety of well-known and less well-known German and translated international writers (*N* = 248) like Alexandre Dumas, Kirsten Boie, Erich Kästner, Ottfried Preussler, Enid Blyton, or Antoine de Saint-Exupeìry. Table 1 shows 10 example books from each corpus. The texts in both corpora were preprocessed using standard python NLP tools, i.e., words were POS-tagged using *treetagger*^2^ and only content words (nouns, verbs, adjectives, and adverbs) were kept for the sentiment analyses using *SentiArt*.

**TABLE 1:** 10 example texts from both corpora.

GLEC-CYL	ChildLex
Andrew Lang. Tales of Troy and Greece	Kirsten Boie. King Kong das Schulschwein
Baronness Orczy. The Scarlet Pimpernel	Michael Ende. Momo
Beatrix Potter. The Tale Of Peter Rabbit	Cornelia Funke. Tintenherz
Edward Stratemeyer. The Rover Boys in the Land of Luck	Martin Klein. Der Geist aus dem Würstchenglas
Jacob Abbott. Cleopatra	Max Kruse. Urmel aus dem Eis
James Matthew Barrie. Peter Pan	Paul Maar. Sams in Gefahr
Louisa May Alcott. Rose in Bloom	Joanne K. Rowling. Harry Potter und der Stein der Weisen
Lyman Frank Baum. The Wonderful Wizard of Oz	Ottfried Preussler. Die kleine Hexe
R M Ballantyne. Away in the Wilderness	Antoine de Saint-Exupeìry. Der Kleine Prinz
Thornton Waldo Burgess. Mrs. Peter Rabbit	Nils Werner. Teddy Brumm

## Methods

### Sentiment Analysis

Dodds et al. (2015) confirmed the Pollyanna hypothesis in large text corpora (e.g., google books, movie subtitles, and twitter) in different languages with a special word-list-based sentiment analysis (“hedonometer”) which selects the most frequent 5–10,000 words only. When analyzing single books, they also used a special method sliding a 10,000-word window through each book and computing the average univariate “happiness score.” Using a “lens” for their hedonometer to obtain a strong signal, they excluded all words for which 3 < happiness score < 7 (i.e., they kept words residing in the tails of each distribution going from 1 to 9).

Our approach using *SentiArt* is different. Instead of using word lists based on human valence ratings – a procedure which presents a number of both methodological and epistemological problems when trying to cross-validate the predictions of a sentiment analysis tool with other human rating data (Hollis et al., 2017; Hofmann et al., 2018) – *SentiArt* is based on VSMs. Using VSMs offers several advantages discussed in previous articles (Jacobs, 2019; Jacobs and Kinder, 2019), such as avoiding these problems and being applicable to any language for which VSMs are publically available (e.g., the >120 VSMs of fasttext^3^).

Unlike most sentiment analysis tools, SentiArt computes a multivariate sentiment analysis offering a dozen affective semantic features (see Table 2) and is empirically validated with diverse experimental data. For example, its affective-aesthetic potential/AAP feature^4^ predicted about 50% of variance in human valence ratings for >2,500 single words and about 45% of variance in “liking” ratings for entire sections from a mystery story (Jacobs and Kinder, 2019). *SentiArt* also achieved 100% accuracy in predicting the sentiment category of 120 excerpts from the Harry Potter books (Jacobs, 2019) outperforming two standard sentiment analysis tools, *VADER* (Hutto and Gilbert, 2014) and *HU-LIU* (Hu and Liu, 2004).

**TABLE 2:** Results of sentiment analyses for GLEC-CYL and childLex.

Feature	Meaning	Computation	GLEC-CYL	ChildLex
Sentences	Average number of sentences / book	NLTK sentence tokenizer	2,402 ± 68	1,116 ± 68.1
Words/sentence	Average number of words / sentence	NLTK word tokenizer	8.0 ± 0.12	5.9 ± 1.4
Tokens	Average number of tokens / book	NLTK word tokenizer	18,589 ± 552	7,351 ± 510
Types	Average number of types / book	NLTK word tokenizer	4,217 ± 105	2,341 ± 106
Word length	Average number of letters / word	NLTK word tokenizer	6.1 ± 0.02	6.5 ± 0.01
Type token ratio	Quotient of average number of types and tokens / book	NLTK word tokenizer	0.26 ± 0.005	0.4 ± 3.005
AAP	Affective-Aesthetic Potential (see Footnote 2)	*SentiArt*, VSM-based using 120 labels	0.29 ± 0.007	0.06 ± 0.004
AAP noun	AAP for nouns only	As AAP	0.21 ± 0.0009	0.22 ± 0.007
AAP verb	AAP for verbs only	As AAP	0.26 ± 0.0005	–.04 ± 0.004
AAP adjective	AAP for adjectives only	As AAP	0.71 ± 0.01	0.17 ± 0.006
AAP adverb	AAP for adverbs only	As AAP	0.50 ± 0.005	0.05 ± 0.003
Anger	Average semantic relatedness between content words and label ‘anger’	*SentiArt*, VSM-based using 1 label only	0.25 ± 0.009	0.26 ± 0.0004
Disgust	As anger	As anger	0.19 ± 0.007	0.15 ± 0.005
Fear	As anger	As anger	0.59 ± 0.008	0.54 ± 0.005
Happiness	As anger	As anger	0.45 ± 0.009	0.53 ± 0.005
Sadness	As anger	As anger	0.30 ± 0.008	0.24 ± 0.005
Surprise	As anger	As anger	0.87 ± 0.009	0.58 ± 0.005
PNR	Ratio of positive/negative words per sentence	As AAP	2.3 ± 0.03	1.5 ± 0.02

*Values are standardized means (*z* values) ± 1 SE.*

Since previous studies showed that the AAP feature fared better at predicting valence or liking ratings than the valence feature, it also computes, AAP will be used in the following analyses. Higher AAP values theoretically indicate a word’s or text’s higher potential for evoking positive affective responses, including aesthetic feelings of liking and beauty. Thus, the AAP was the most important feature in a recent empirical study showing that human beauty ratings for single words can perfectly be classified via machine learning on the basis of a total of eight quantitative word features (Jacobs, 2017). In the following analyses, the AAP feature is complemented by six discrete emotion features (anger, disgust, fear, happiness, sadness, and surprise) based on classic emotion theories (for review, see Westbury et al., 2015). *SentiArt* computes these six features (similarly to AAP) via a VSM-based procedure using single labels (e.g., the word “happiness”) instead of sets comprising 60 items. Thus, the “happiness” score of a sentence, for example, corresponds to the average semantic relatedness between each content word and the word “happiness,” as computed via the cosine between the corresponding word vectors.

From a psychological, reader response, or neurocognitive poetics perspective, two questions are important. First, on which unit(s) of text a normal reader bases her affective-aesthetic appreciation of an entire book or book chapter: words, sentences, paragraphs, or perhaps pages? The answer to this first question is unknown and a constant challenge to researchers in the emerging fields of neurocognitive poetics (Jacobs, 2015a; Willems and Jacobs, 2016; Nicklas and Jacobs, 2017). For practical reasons, when running empirical studies on whole books, the sentence has appeared to be the smallest viable unit. For example^5^, Jockers (2017) had four readers rate each sentence’s valence of several contemporary novels and found correlations ranging from.52 to.83 between the predictions of his sentiment analysis tool (i.e., the word list based *Syuzhet*) and the readers’ ratings, depending on the novel. Other researchers had readers rate the valence of “story sections” (corresponding roughly to paragraphs; Lehne et al., 2015) or each sentence of book chapters (Jacobs et al., 2020). We are not aware of studies of entire books using readers’ valence rating on word level and thus follow Jockers in selecting the sentence as the basic unit. Note, however, for Bestgen’s (1994) seminal study, readers rated about 40% of the unique content words (types) of certain shorter texts (e.g., Hans Christian Andersen’s “The little match girl”), albeit not in the story context.

A sentence-based procedure of course raises the second question whether the average word valence is the optimal estimate for a sentence’s valence. Bestgen’s (1994, Table 2) correlational data – the most informative for answering this question, as far as we can tell – suggest that lexical (word) valence predicted between 30 and 60% of the variance in (average) sentence valence, while (average) sentence valence predicted between 60 and 70% of the variance in text valence, depending on the text.

Given these results, it seemed most promising to compute the mean valence (or, in our case, AAP) averaged across all content words (nouns, verbs, adjectives, and adverbs) of a sentence. In addition, we also computed mean AAP sentence values based on distinct word types only, e.g., nouns or adjectives. This is because it is so far unknown to what extent different word types (lexico-grammatical information) may contribute to the emotional evaluation of a sentence. The data by Lüdtke and Jacobs (2015) suggested non-linear interactive effects between the valence of nouns and adjectives on emotional evaluations of simple short declarative sentences (e.g., “The grandpa is lonely”). They showed that negative adjectives dominated supralexical evaluation, which can be interpreted as a sort of negativity bias. Since so far, no empirical follow-up studies have investigated more complex sentences or the influence of other word types (e.g., verbs and adverbs), we do not know whether Lüdtke and Jacobs’ findings may generalize to complex sentences. This is important because the bulk of sentences contained in our literature corpora are complex ones. In addition to the overall mean sentence AAP, and mean noun-, verb-, adjective- and adverb-based AAP, we also computed the ratio between the frequency of positive (AAP value > 0) and negative words (AAP value < 0) per sentence (the PNR). The PNR allows answering the question whether positive words were used more often in a book than negative words, as hypothesized by Boucher and Osgood (1969). Finally, we computed the semantic relatedness of each content word in a sentence with each of the six basic emotions (i.e., anger, disgust, fear, happiness, sadness, and surprise) and the corresponding mean per sentence. Thus, for each sentence of every book 12 affective semantic features went into the present sentiment analyses (see Table 2).

## Results and Discussion

### Study 1: Cross-Validation of *SentiArt* With Human Rating Data From the kidBAWL

**FIGURE 1:**
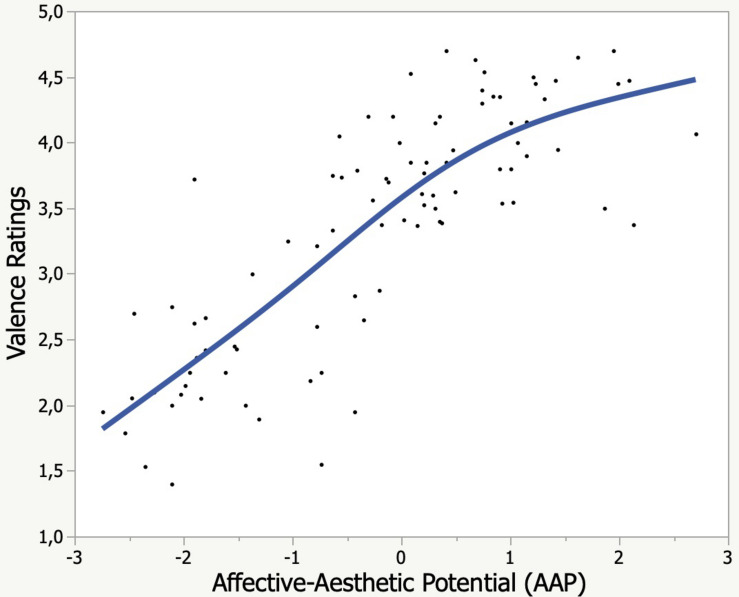
Rated word valence as a function of computational affective-aesthetic potential (AAP).

The predictive validity of the AAP feature computed by *SentiArt* was tested with the kidBAWL valence rating data from Sylvester et al.’s (2016) Experiment 1, where six to 12 years old children read a subset of 90 words from the kidBAWL and judged the word’s valence on a 5-point scale (very unpleasant – unpleasant – neither unpleasant nor pleasant – pleasant – very pleasant). The results of the cross validation shown in Figure 1 support those of the aforementioned studies, establishing a good empirical predictive validity of *SentiArt* for human rating data, yielding an *R*^2^_*adj*_ = 0.68 (logistic fit; linear fit: *R*^2^_*adj*_ = 0.65). Together with the findings of previous studies (Westbury et al., 2015; Hollis et al., 2017; Hofmann et al., 2018; Jacobs, 2019; Jacobs and Kinder, 2019) this offers even more evidence supporting the validity of VSM-based sentiment analysis tools which, in contrast to word list based tools, cannot be criticized for the aforementioned epistemological or psychometric problems. Having shown the validity of *SentiArt* with rating data from children of age 7 to 12, we now proceed with the computational text analyses.

### Study 2: Sentiment Analysis of Porter’s (1915) “Pollyanna Grows Up”

**FIGURE 2:**
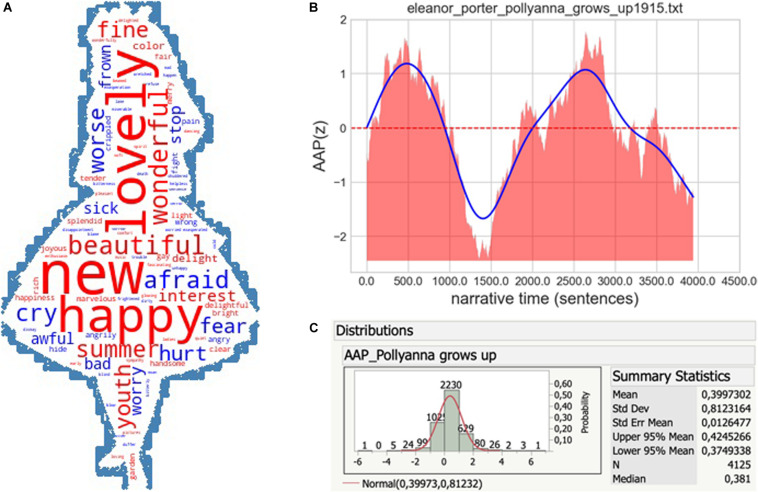
**(A–C)** Wordcloud, emotional (narrative) time series and affective-aesthetic potential (AAP) distribution for “Pollyanna grows up.” The blue curve in **(B)** represents the smoothed average, the dotted red line the zero border.

Before we proceed to test whether CYL at large indeed exhibits the “Pollyanna” effect (as suggested by preliminary research), we think it is quite natural to ask whether the effect is exhibited by the very book whose protagonist became emblematic of it. The following computational data based on *SentiArt’s* AAP feature suggest a “yes” answer. The wordcloud in Figure 2A gives a first idea why the book is – on average – more positive than negative. The cloud summarizes data of the 1,000 most positive and 1,000 most negative words in the book and positive words like NEW or LOVELY (the words with the highest frequency of occurrence among the 2,000; *N* = 53 and 43, respectively) clearly dominate negative ones like AFRAID or CRY (*N* = 25 and 22, respectively). More detailed evidence suggesting that the Pollyanna hypothesis is borne out is shown in Figures 2B,C. The “emotional time series” in Figure 2B shows a profile which resembles the typical “man in hole” emotional arc profile (Vonnegut, 1981; Reagan et al., 2016), except for the final fall. The fact that most of the area of the smoothed curve lies above the zero line indicates an overall positive AAP. Figure 2C corroborates the positivity bias with histogram data which have a mean AAP value of 0.4 for 4,125 sentences^6^. Although showing the effect in the very book that coined its name may not come as a big surprise, this finding yields another, if slightly informal, type of validation of the said effect and thus is a good start for the upcoming analyses of the 372 GLEC-CYL and 500 childLex books.

### Study 3: Sentiment Analysis of GLEC-CYL and ChildLex

Here we computed the 12 affective semantic features outlined in Table 2 for our two corpora using *SentiArt* together with other text features indicating style such as type token ratio (Jacobs, 2018a). The mean hit-rate or coverage (i.e., the overlap between the VSM’s vocabulary and all content words in all books) was 98% for GLEC-CYL and 88% for childLex.

The global statistics for the books from two CYL corpora given in Table 2 can be summarized as follows. On average, the English books are longer than the German ones, having more sentences and more words per sentence. Note, however, that these comparisons are only suggestive and cannot be generalized without further investigations given the enormous differences in publication period or number of different authors between the two corpora.

Regarding the sentiment analysis, GLEC-CYL books generally adhere to the Pollyanna principle exhibiting a positivity bias for all AAP values and a ratio of positive/negative words per sentence (PNR) of 2.3 per sentence, i.e., on average there are clearly more positive words than negative ones in a sentence. At the level of semantic relations with discrete emotion words, GLEC-CYL books are dominated by surprise, fear, and happiness, while sadness, anger and disgust play minor roles. With regard to the abovementioned issue of a possible negativity bias due to noun–adjective interactions (Lüdtke and Jacobs, 2015) the GLEC-CYL data rather indicate, at least on average, a positivity bias, since both “AAP noun” and “AAP adjective” features are positive.

Just as the English corpus, the books from childLex also generally adhere to the Pollyanna principle. Note, though, that the affective semantic feature values from the two corpora are not directly comparable, since they stem from different VSMs. Similarly to GLEC-CYL, childLex exhibits a general positivity bias^7^ – except for AAP verb – and with a PNR of 1.5 per sentence a clear signal of more positive words at the sentence level. Regarding discrete emotion words, we find the same pattern as for GLEC-CYL with surprise, fear, and happiness dominating in childLex books, while sadness, anger and disgust play minor roles. Again, there is no general indication of a possible negativity bias due to noun–adjective interactions, both being congruently positive on average.

**FIGURE 3:**
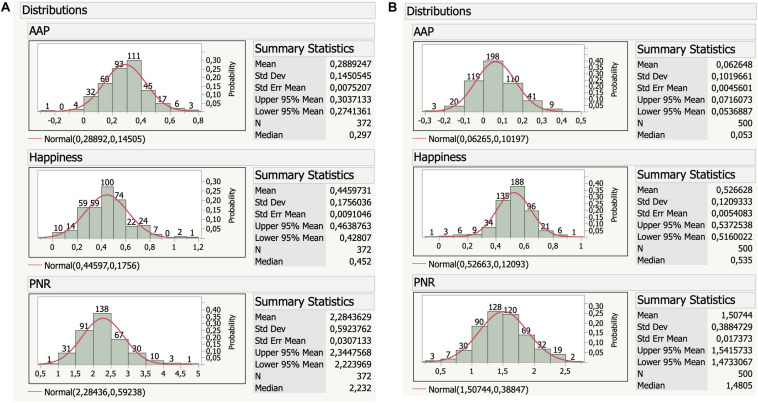
**(A,B)** Affective-aesthetic potential (AAP), happiness score and ratio of positive/negative words per sentence (PNR) distributions for GLEC-CYL (left) and childLex (right).

The distributional data in Figure 3 show that a single book in GLEC-CYL had an overall negative AAP (Beatrix Potter’s “The Story of Miss Moppet”), while in childLex 142 books exhibit an overall negativity bias (∼30%). Again, the results for the very homogeneous GLEC-CYL in which 341/372 books stem from only nine different authors cannot directly be compared, though, to those of childLex. Thus, we can only propose two heuristic hypotheses: there might be (a) a more pronounced positivity bias in English CYL when compared to German CYL; (b) a trend toward a less pronounced positivity bias from 19^*th*^ century to contemporary CYL. Both hypotheses (and possible interactions) need to undergo further testing in future studies.

What is relatively safe to formulate as a testable hypothesis is that in both corpora readers have a higher probability of positive feelings associated with surprise than for negative feelings associated with disgust. Readers of books from both corpora also face a theoretically high probability of experiencing thoughts or feelings associated with fear. According to our sentiment analyses, this probability would be highest for the following three books from the GLEC-CYL corpus: James Matthew Barrie’s “Tommy and Grizel,” Louisa May Alcott’s “Pauline’s Passion and Punishment,” and Thornton Waldo Burgess’ “Lightfoot the Deer” which all showed ‘fear’ scores of >1. In childLex, it would be: Knister’s (Ludger Jochmann), “Hexe Lilli und der verflixte Gespensterzauber,” as well as Sabine Neuffer’s “Lukas und Felix werden Freunde” and Kirsten Boie’s, “King Kong das Krimischwein” (fear score > 0.9). When discussing these findings, incorporating contextual information is crucial. It is thus an open empirical question whether – within the appropriate reading context (paragraph and chapter), and not in isolation– a single sentence from Barrie’s book like “Young man, I fear you are doomed,” for which the relatively highest fear score was computed, really induces higher, “fear” ratings than other sentences, or whether, it is rather the book as a whole that has a higher probability of being associated with fear feelings(when compared to other books). In addition, the feeling that a single sentence may induce will depend on a mix of different scores, e.g., to what extent a word having a high fear score is in the company of words having high happiness or sadness scores, and as well on the mean AAP value. Moreover, also the question whether mean AAP will be the best approximation of sentence AAP or whether some kind of word type by valence interaction has to be taken into account (cf. Lüdtke and Jacobs, 2015), have to be answered by future studies. We believe that predictive modeling studies using advanced computational text analysis tools like *SentiArt* (e.g., Jacobs, 2017; Xue et al., 2019, 2020) are not the only but surely a very promising way of finding out which of these possibilities come near to reality, which, of course, also involves effects of the preceding and following context, and of reader personality factors such as mood (Lüdtke et al., 2014; Jacobs et al., 2016b).

Thus, being based on computational models, the present sentiment analyses, like others, cannot provide a necessity, but only a sufficiency analysis, i.e., they are a tool for quantitatively predicting how things could be, if certain conditions hold. Whether this corresponds to reality must be determined via adequate empirical testing which then can inform the improvement of computational sentiment analysis tools, e.g., by indicating that the present six discrete emotion scores are not sufficient for good reader response predictions and should be augmented (or replaced) by other scores. It should be noted, though, that apart from the aforementioned behavioral studies cross-validating predictions from *SentiArt* there is also neuroimaging evidence indicating that text passages from the Harry Potter books which have a high theoretical “fear” potential can indeed activate brain regions associated with fear induction (Hsu et al., 2015).

## Conclusion, Limitations, and Outlook

Despite differences in the computational methods, the present sentiment analysis results support the findings reported by Dodds et al. (2015), showing that also international classical and contemporary CYL generally exhibits the Pollyanna principle as hypothesized by Boucher and Osgood (1969). Both an English corpus from the 19^*th*^ century with only 25 different authors and a contemporary German corpus with >200 different authors clearly show a positivity bias, not only in a single text feature (as, e.g., in Dodds et al.’s univariate sentiment analysis), but in a variety of features such as AAP, AAP noun, happiness, or PNR. Together with those of previous cross-validation studies (e.g., Jacobs and Kinder, 2019) the results of the present Study 1 are promising, accounting for almost 70% of valence ratings. However, at the same time, they still leave ∼30% of variance unaccounted for. This could be due to the unknown experiential/embodied part of affective semantics that cannot be captured by distributional semantics models like the VSMs used here (Jacobs et al., 2016a), or, of course to other possible limitations of *SentiArt*, e.g., regarding the choice of labels. When applying lexical sentiment values to larger units of text (i.e., sentences and chapters) the amount of unaccounted variance can, but must not necessarily, increase nonlinearly, as can be inferred from the correlations obtained by the aforementioned studies by Bestgen (1994) and Jockers (yielding *R*^2^ values for sentences between 0.27 and 0.7, depending on the text). There is definitely a long way to go before a fuller understanding of the processes underlying readers’ affective-aesthetic text evaluation is achieved. Combined efforts complementing quantitative (“distant”) digital humanities with close reading studies are necessary, as are greater efforts in developing adequate training corpora, VSMs and empirical designs combining direct offline with indirect online methods of scientific studies of literature (Dixon and Bortolussi, 2015; Jacobs, 2015b; Kuiken, 2015). Reporting on a variety of distinct measures for gauging sentiment and emotion for “positivity” in young readers’ books, our study has shown that a widely discussed phenomenon such as the Pollyanna effect can – and in fact should – undergo further nuanced theoretical and computational modeling. In addition to the more “standard” univariate measures, our unsupervised multivariate approach theoretically allows for a more nuanced modeling of aesthetic emotions in literature reception. As these represent readers’ interaction with the “poetic form” (Jakobson, 1960), their incorporation offers a more refined account of the affective ecology of literary reading, and thus a deeper grasp of “positivity” encoded in our cultural heritage.

## Data Availability Statement

The raw data supporting the conclusions of this article will be made available by the authors, without undue reservation.

## Author Contributions

AJ designed the study, analyzed the data with advice from all authors, and drafted the manuscript. All authors contributed to the final version of the manuscript.

## Conflict of Interest

The authors declare that the research was conducted in the absence of any commercial or financial relationships that could be construed as a potential conflict of interest.

## Footnotes

2 https://www.cis.uni-muenchen.de/∼schmid/tools/TreeTagger/

3 https://fasttext.cc/docs/en/pretrained-vectors.html

4 AAP refers to the average semantic relatedness between each word in a text and *m* positive labels (*lpos*_1-60 = affection, amuse,…, unity) minus the average relatedness between each word and *n* negative labels (*lneg*_1-60 = abominable,…, ugly). The labels were published in earlier papers (Jacobs, 2017, 2018b). For English, the semantic relatedness between a test word and each of the 120 labels is computed using the *GLEC* vector space model/VSM (Jacobs, 2018b) which was created applying the *fasttext* algorithm (https://fasttext.cc/) to the ensemble of texts in *GLEC*. The final model contained a 500d *skipgram* vector for each word from *GLEC* ordered by frequency of occurrence. The cosine of two word vectors gives the semantic relatedness value. For German, the VSM was the 300d skipgram SDEWAC/subtlex model (see Jacobs and Kinder, 2019; Table 1).

5 http://www.matthewjockers.net/2019/03/18/new-network-viz/

6 A *z*-test established that the mean value of 0.4 is significantly greater than 0 (*z* = 31.7, *p* < 0.0001).

7 A *z*-test established that the mean value of 0.06 is significantly greater than 0 (*z* = 14.0, *p* < 0.0001).

## References

[B1] Ashby F. G. Isen A. M. (1999). A neuropsychological theory of positive affect and its influence on cognition. *Psychol. Rev.* 106:529. 10.1037/0033-295x.106.3.529 10467897

[B2] Bestgen Y. (1994). Can emotional valence in stories be determined from words? *Cogn. Emot.* 8 21–36. 10.1080/02699939408408926

[B3] Boucher J. Osgood C. E. (1969). The pollyanna hypothesis. *J. Verb. Learn. Verb. Behav.* 8: 1–8. 10.1016/S0022-5371(69)80002-2

[B4] Dixon P. Bortolussi M. (2015). Measuring literary experience: comment on Jacobs. *Sci. Study Lit.* 5 178–182. 10.1075/ssol.5.2.03dix

[B5] Dodds P. S. Clark E. M. Desu S. Frank M. R. Reagan A. J. Williams J. R. (2015). Human language reveals a universal positivity bias. *Proc. Natl. Acad. Sci. U.S.A.* 112: 2389-2394. 10.1073/pnas.1411678112 PMC4345622 25675475

[B6] Forgas J. P. (2013). Don’t worry, be sad! On the cognitive, motivational, and inter-personal benefits of negative mood. *Curr. Dir. Psychol. Sci*. 22, 225–232.

[B7] Greene C. (2017). Introducing the corpus of the Canon of Western Literature: a corpus for culturomics and stylistics. *Lang. Lit.* 26 282–299. 10.1177/0963947017718996

[B8] Hofmann M. J. Jacobs A. M. (2014). Interactive activation and competition models and semantic context: from behavioral to brain data. *Neurosci. Biobehav. Rev.* 46 85–104. 10.1016/j.neubiorev.2014.06.011 24992217

[B9] Hofmann M. J. Kuchinke L. (2015). “Anything is good that stimulates thought” in the hippocampus Comment on “The quartet theory of human emotions: an integrative and neurofunctional model” by S. Koelsch et al. *Phys. Life Rev.* 13 58–60. 10.1016/j.plrev.2015.04.007 25912771

[B10] Hofmann M. J. Biemann C. Westbury C. Murusidze M. Conrad M. Jacobs A. M. (2018). Simple co-occurrence statistics reproducibly predict association ratings. *Cogn. Sci*. 42 1–26. 10.1111/cogs.12662 30098213

[B11] Hollis G. Westbury C. Lefsrud L. (2017). Extrapolating human judgments from skip- gram vector representations of word meaning. *Q. J. Exp. Psychol*. 70 1–45. 10.1080/17470218.2016.1195417 27251936

[B12] Hsu C. T. Jacobs A. M. Citron F. Conrad M. (2015). The emotion potential of words and passages in reading Harry Potter - An fMRI study. *Brain Lang.* 142 96–114. 10.1016/j.bandl.2015.01.011 25681681

[B13] Hu M. Liu B. (2004). “Mining and summarizing customer reviews,” in *Proceedings of the Tenth ACM SIGKDD International Conference on Knowledge Discovery and Data Mining*, eds Kim W. Kohavi R. (Washington, DC: ACM Press), 168–177. 10.1145/1014052.1014073

[B14] Hutto C. J. Gilbert E. E. (2014). “VADER: a parsimonious rule-based model for sentiment analysis of social media rext,” in *Proceedings of the Eighth International Conference on Weblogs and Social Media (ICWSM-14).* Ann Arbor, MI.

[B15] Jacobs A. M. (2015a). Neurocognitive poetics: methods and models for investigating the neuronal and cognitive-affective bases of literature reception. *Front. Hum. Neurosci*. 9:186. 10.3389/fnhum.2015.00186 25932010 PMC4399337

[B16] Jacobs A. M. (2015b). The scientific study of literary experience: sampling the state of the art. *Sci. Study Lit.* 5 139–170. 10.1075/ssol.5.2.01jac

[B17] Jacobs A. M. (2017). Quantifying the beauty of words: a neurocognitive poetics perspective. *Front. Hum. Neurosci.* 11:622. 10.3389/fnhum.2017.00622 29311877 PMC5742167

[B18] Jacobs A. M. (2018a). Neuro-Cognitive poetics and computational stylistics. *Sci. Study Lit*. 8 164–207. 10.1075/ssol.18002.jac

[B19] Jacobs A. M. (2018b). The gutenberg english poetry corpus: exemplary quantitative narrative analyses. *Front. Digit. Humanit.* 5:5 10.3389/fdigh.2018.00005

[B20] Jacobs A. M. (2019). Sentiment analysis for words and fiction characters from the perspective of computational (Neuro-)Poetics. *Front. Robot.* 6:53 10.3389/frobt.2019.00053 PMC7805775 33501068

[B21] Jacobs A. M. Kinder A. (2019). Computing the affective-aesthetic potential of literary texts. *Artif. Intell.* 1 11–27. 10.3390/ai1010002

[B22] Jacobs A. M. Võ.M. L.-H. Briesemeister B. B. Conrad M. Hofmann M. J. Kuchinke L. (2015). 10 years of BAWLing into affective and aesthetic processes in reading: what are the echoes? *Front. Psychol.* 6:714. 10.3389/fpsyg.2015.00714 26089808 PMC4452804

[B23] Jacobs A. M. Hofmann M. J. Kinder A. (2016a). On elementary affective decisions: to like or not to like, that is the question. *Front. Psychol.* 7:1836. 10.3389/fpsyg.2016.01836 27933013 PMC5122311

[B24] Jacobs A. M. Lüdtke J. Aryani A. Meyer-Sickendiek B. Conrad M. (2016b). Mood-empathic and aesthetic responses in poetry reception: a model-guided, multilevel, multimethod approach. *Sci. Study Lit.* 6 87–130. 10.1075/ssol.6.1.06jac

[B25] Jacobs A. M. Usée F. Lüdtke J. (2020). Predicting readers’ sentiments with Sentiart. (in preparation).

[B26] Jakobson R. (1960). “Closing statement: linguistics and poetics,” in *Style in language* ed. Sebeok T. A. (Cambridge, MA: The MIT Press), 350–377.

[B27] Jockers M. (2017). *Introduction to the Syuzhet Package.* Available online at: https://cran.r-project.org/web/packages/syuzhet/vignettes/syuzhet-vignette.html (accessed August 29, 2020).

[B28] Kuchinke L. Jacobs A. M. Grubich C. Võ M. L.-H. Conrad M. Herrmann M. (2005). Incidental effects of emotional valence in single word processing: an fMRI study. *Neuroimage* 28 1022–1032. 10.1016/j.neuroimage.2005.06.050 16084739

[B29] Kuiken D. (2015). The implicit erasure of “literary experience” in empirical studies of literature: comment on “the scientific study of literary experience: sampling the state of the art” by Arthur Jacobs. *Sci. Study Lit*. 5 171–177. 10.1075/ssol.5.2.02kui

[B30] Lehne M. Engel P. Rohrmeier M. Menninghaus W. Jacobs A. M. Koelsch S. (2015). Reading a suspenseful literary text activates brain areas related to social cognition and predictive inference. *PLoS One* 10:e0124550. 10.1371/journal.pone.0124550 25946306 PMC4422438

[B31] Lüdtke J. Jacobs A. M. (2015). The emotion potential of simple sentences: additive or interactive effects of nouns and adjectives? *Front. Psychol.* 6:1137. 10.3389/fpsyg.2015.01137 26321975 PMC4531214

[B32] Lüdtke J. Meyer-Sickendiek B. Jacobs A. M. (2014). Immersing in the stillness of an early morning: testing the mood empathy hypothesis in poems. *Psychol. Aesthet. Creat. Arts* 8 363–377. 10.1037/a0036826

[B33] Nicklas P. Jacobs A. M. (2017). Rhetorics, neurocognitive poetics and the aesthetics of adaptation. *Poetics Today* 38 393–412.

[B34] Willems R. Jacobs A. M. (2016). Caring about Dostoyevsky: the untapped potential of studying literature. *Trends Cogn. Sci*. 20 243–245. 10.1016/j.tics.2015.12.009, 26809726

[B35] Porter E. (1915). *Pollyanna Grows Up.* Boston: The Page Company.

[B36] Reagan A. J. Mitchell L. Kiley D. Danforth C. D. Dodds P. S. (2016). The emotional arcs of stories are dominated by six basic shapes. *EPJ Data Sci.* 5:31.

[B37] Rowling J. K. (1997). *Harry Potter and the Philosopher’s Stone.* London: Bloomsbury.

[B38] Schroeder S. Würzner K. M. Heister J. Geyken A. Kliegl R. (2015). childLex: a lexical database of German read by children. *Behav. Res. Methods* 47 1085–1094. 10.3758/s13428-014-0528-1 25319039

[B39] Sylvester T. Braun M. Schmidtke D. Jacobs A. M. (2016). The Berlin affective word list for children (kidBAWL): exploring processing of affective lexical semantics in the visual and auditory modalities. *Front. Psychol*. 7:969. 10.3389/fpsyg.2016.00969 27445930 PMC4928334

[B40] Unkelbach C. von Hippel W. Forgas J. P. Robinson M. D. Shakarchi R. J. Hawkins C. (2010). Good things come easy: subjective exposure frequency and the faster processing of positive information. *Soc. Cogn.* 28 538–555. 10.1521/soco.2010.28.4.538

[B41] Vonnegut K. (1981). *Palm Sunday.* NewYork, NY: Rosetta Books LLC.

[B42] Westbury C. Keith J. Briesemeister B. B. Hofmann M. J. Jacobs A. M. (2015). Avoid violence, rioting, and outrage; approach celebration, delight, and strength: using large text corpora to compute valence, arousal, and the basic emotions. *Q. J. Exp. Psychol.* 68 1599–1622. 10.1080/17470218.2014.970204 26147614

[B43] Xue S. Lüdtke J. Sylvester T. Jacobs A. M. (2019). Reading Shakespeare Sonnets?: combining quantitative narrative analysis and predictive modeling — an eye tracking study. *J. Eye Mov. Res*. 12 1–16. 10.16910/jemr.12.5.2 PMC7968390 33828746

[B44] Xue S. Jacobs A. M. Lüdtke J. (2020). What is the difference? rereading Shakespeare’s Sonnets - an eye tracking study. *Front. Psychol.* 11:421. 10.3389/fpsyg.2020.00421 32273860 PMC7113389

